# Laser-Ablative Synthesis of Stable Aqueous Solutions of Elemental Bismuth Nanoparticles for Multimodal Theranostic Applications

**DOI:** 10.3390/nano10081463

**Published:** 2020-07-26

**Authors:** Julia C. Bulmahn, Gleb Tikhonowski, Anton A. Popov, Andrey Kuzmin, Sergey M. Klimentov, Andrei V. Kabashin, Paras N. Prasad

**Affiliations:** 1 Department of Chemistry and Institute for Lasers, Photonics, and Biophotonics, University at Buffalo, The State University of New York, Buffalo, NY 14260, USA; juliabul@buffalo.edu (J.C.B.); ankuzmin@buffalo.edu (A.K.); 2 Bionanophotonic Lab., Institute of Engineering Physics for Biomedicine (PhysBio), National Nuclear Research University MEPHI, 115409 Moscow, Russia; gtikhonowski@gmail.com (G.T.); aapopov@mephi.ru (A.A.P.); kliment-61@mail.ru (S.M.K.); 3 LP3, Aix Marseille University, CNRS, 13288 Marseille, France

**Keywords:** bismuth (Bi) nanoparticles, nanosheets, laser ablation in liquids, radiotherapy, phototherapy, nanomedicine

## Abstract

Elemental bismuth (Bi) nanoparticles (NPs), with the high atomic density of the Bi nuclei, could serve as efficient targeted agents for cancer treatment, with applications such as contrast agents for computed tomography (CT) imaging, sensitizers for image-guided X-ray radiotherapy, and photothermal therapy. However, the synthesis of elemental Bi NPs suitable for biological applications is difficult using conventional chemical routes. Here, we explore the fabrication of ultrapure Bi-based nanomaterials by femtosecond laser ablation from a solid Bi target in ambient liquids and characterize them by a variety of techniques, including TEM, SEM, XRD, FTIR, Raman, and optical spectroscopy. We found that laser-ablative synthesis using an elemental Bi solid target leads to the formation of spherical Bi NPs having the mean size of 20–50 nm and a low size-dispersion. The NPs prepared in water experience a fast (within a few minutes) conversion into 400–500 nm flake-like nanosheets, composed of bismuth subcarbonates, (BiO)_2_CO_3_ and (BiO)_4_CO_3_(OH)_2_, while the NPs prepared in acetone demonstrate high elemental stability. We introduce a procedure to obtain a stable aqueous solution of elemental Bi NPs suitable for biological applications, based on the coating of Bi NPs prepared in acetone with Pluronic^®^ F68 and their subsequent transfer to water. We also show that the laser-synthesized elemental Bi NPs, due to their vanishing band gap, exhibit remarkable absorption in the infrared range, which can be used for the activation of photothermal therapy in the near IR-to-IR window with maximum optical transparency in biological media. Exempt of any toxic synthetic by-products, laser-ablated elemental Bi NPs present a novel appealing nanoplatform for combination image-guided photoradiotherapies.

## 1. Introduction

Nanomaterials with high atomic numbers (Z) have demonstrated their ability to act as efficient sensitizers of radiotherapy (RT) [1,2,3]. When these high-Z elements are irradiated with X-rays, the result is a high local ionization effect leading to DNA strand breaks and ultimately enhancing the efficacy of RT. Off target damage is also limited, as the range of this effect is less than 10 nm; consequently, lower doses of RT are needed to achieve the desired effects [4]. 

From a biomedical prospective, bismuth (Z = 83) has several advantages over other high atomic number elements. First, Bi has the highest atomic number of all non-radioactive elements leading to excellent radiosensitization properties. Bi also has a very high X-ray attenuation coefficient (5.74 cm^2^/g at 100 keV), which leads to its usage in high contrast computed tomography (CT) imaging [5]. Additionally, as a metal, elemental Bi strongly absorbs light over a broad spectral range extending to IR, which allows the resulting photothermal heating of elemental Bi nanoparticles by an IR light to be used for photoacoustic imaging as well as for photothermal therapy, which can synergistically enhance radiotherapy [3]. It is also important to note that Bi demonstrates low toxicity, good biocompatibility, and increased cost effectiveness in comparison to other high-Z elements [2]. Furthermore, Bi can also be easily eliminated from the body due to its favorable reactivity and dissolution properties [6,7]. Bi-based compounds have already been successfully explored in CT imaging and radiotherapy [2,3], as well as been used in off-the-shelf medications (e.g., Pepto-Bismol [8]), which confirms their relative safety. 

The fabrication of Bi-based nanoparticles (NPs) typically requires chemical pathways, [9,10,11] but these methods are not fully compatible with stringent requirements of biological systems due to the necessity of using toxic solvents and substances. Based on physical mechanisms of nanostructure formation, laser ablation in liquids is free of limitations of chemical methods, offering a solution to the toxicity problems [12,13]. The laser-ablative approach profits from a natural production of nanoclusters under the interaction of pulsed laser radiation with a solid target, which are then released to an ambient liquid to form a colloidal nanoparticle solution [14,15,16]. When ablated in a pure ambient (deionized water, ethanol, acetone, etc.), the formed NPs are exempt of any toxic contamination, which opens up avenues for the synthesis of different nanomaterials for biological use, including Au NPs [15,16,17,18], Si NPs [19,20], TiN NPs [21], and Sm [22]. Methods of laser ablation have already been explored for the fabrication of Bi-based nanomaterials [23,24,25,26,27]. However, laser ablation in water has yielded only stable Bi compound NPs (oxides, carbonates, etc.), which cannot provide the maximum local concentration of Bi nuclei in comparison to elemental Bi, as a high concentration of Bi would reduce the needed dose level for radiotherapy and would also enhance the contrast for imaging. Furthermore, the transfer of elemental Bi NPs prepared in organic solutions when transferred to water typically suffers from a prompt oxidation of NPs and destabilization of solutions. These oxidized Bi compounds, having a wide bandgap, do not significantly absorb to effect sufficient photothermal therapy using IR light. 

Here, we investigate conditions of synthesis and properties of Bi-based NPs by using methods of femtosecond laser ablation. We report the fabrication of stable aqueous solutions of ultrapure elemental Bi NPs by laser ablation in acetone, followed by their coating with Pluronic^®^ F68 and transfer to water in unchanged form. This suggests that a proper surface modification of laser-synthesized Bi NPs is a plausible method to prepare stable, biocompatible, and pure Bi NPs for applications in nanomedicine.

## 2. Materials and Methods

### 2.1. Synthesis of Nanoparticles

**Figure 1:**
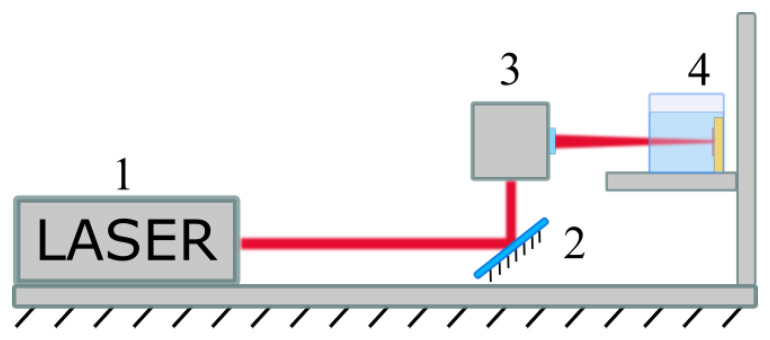
Schematic representation of the experimental setup for laser ablation. 1. a laser unit; 2. a mirror; 3. a galvanometric scanner and a focusing F-theta lens; 4. the ablation chamber with the Bi target submerged in liquid.

NPs were synthesized by ultrashot (fs) laser ablation of the bismuth (Bi) target (GoodFellow, Coraopolis, PA, USA, purity 99.999%) in deionized water (18.2 MΩ cm at 25 °C) or technical grade acetone, under ambient conditions. A schematic of the experimental setup is shown in Figure 1. The Bi target was fixed vertically on the wall of a quartz vessel filled with 40 mL of a liquid. A 3-mm-diameter beam from a Yb:KGW laser (1030 nm wavelength, 270 fs pulse duration, 30 µJ pulse energy, 100 kHz repetition rate; TETA 10 model, Avesta, Moscow, Russia) was focused by a 100-mm F-theta lens on the surface of the target, through a side wall of the ablation vessel. The thickness of the liquid layer along the laser beam was 6 mm. The focusing conditions were set to obtain maximum productivity from the ablation process (defined as ablated mass per duration of ablation) individually for each solvent. The laser beam was moved over a 20 × 20 mm area on the surface of the target, with 4000 mm/s speed using a galvanometric scanner. This was done to avoid drilling of a hole in the target and to maximize the nanoparticle (NP) yield. The duration of each laser ablation experiment was 15 min. The target and the ablation chamber were cleaned after each ablation experiment, by using an ultrasonication step in acetone, followed by ultrasonication in water, thorough rinsing in ultrapure water and, finally, drying under ambient conditions.

### 2.2. Surface Modification of Bi NPs

Bi NPs ablated in acetone were successfully transferred to water using a surface coating of Pluronic^®^ F68 (Sigma-Aldrich, St. Louis, MO, USA). To achieve this surface coating, 50 mg of Pluronic ^®^ F68 was added to 10 mg of Bi NPs in acetone, and this was vortexed for 30 s to dissolve the polymer. This solution was then evaporated to dry using moderate air flow and resuspended in 1 mL distilled water with sonication. The resulting dispersion was centrifuged 10 min at 3000 RCF and the supernatant was discarded to remove any excess polymer. The resulting pellet was resuspended in 1 mL deionized water to produce a stable brown dispersion. The NPs were stored at RT for further use.

### 2.3. Characterization of Nanoparticles

Morphology, structure, size and composition of NPs and nanosheets were characterized by transmission electron microscopy (TEM) using a JEM-2010 microscope (JEOL USA, Inc., Peabody, MA, USA) at an acceleration voltage of 200 kV or scanning electron microscopy (SEM) using a MAIA 3 microscope (Tescan, Czech Republic) operating at 0.1–30 kV accelerating voltage. Samples for electron microscopy were prepared by dropping 10 μL of the NPs solution onto a formvar-coated copper grid (for TEM) or cleaned crystalline silicon substrate (for SEM), with subsequent drying at ambient conditions. The hydrodynamic diameter and ζ–potential measurements were performed using a 90Plus zeta sizer (Brookhaven Instruments, Holtsville, NY, USA). The powder X-ray diffraction (XRD) patterns were recorded by a Rigaku Ultima IV X-Ray Diffractometer (Rigaku, Tokyo, Japan), using Cu Kα radiation (λ = 0.15418 nm). The 2θ angle of the XRD patterns was recorded at a scanning rate of 2 °/min. The Fourier-transform infrared (FTIR) spectra were recorded using a Spectrum II FTIR spectrophotometer with a micro-Attenuated Total Reflectance sampling accessory (Perkin Elmer, Waltham, MA, USA). Samples were placed on a 2-mm diamondwindow and spectra were recorded in the wavenumber range of 400–4000 cm^−1^ with a resolution of 4 cm^−1^. The force gauge reading was 50 units. ATR correction was applied. Raman spectra were measured using a DXR2 Raman microscopy system (Thermo Fisher Scientific, Madison, WI) with a red laser source (ROUSB-633-PLR-70-1, Ondax, CA, USA) and a Plan N oil immersion 100x (Na = 1.25) objective lens (Olympus, Japan). The following measurement parameters were used: 7 mW Continuous Wave power on the sample, 1 s of accumulation time, and 50 µm of confocal pinhole diameter. Raman spectra were collected and processed by the OMNIC software for dispersive Raman (Thermo Fisher Scientific, Madison, WI, USA). Water dispersed NPs were sealed between a microscope slide and a cover slip, while acetone-dispersed NPs were sealed in a capillary tube (ø 300 µm, 10 mm).

### 2.4. Photothermal Gradient Measurement

The temperature distribution dynamics induced by the nanomaterials under 808 nm laser irradiation was monitored by a thermal imaging camera FLIR A600 (FLIR Systems, Wilsonville, OR, USA). For thermal gradient dynamics measurements, all samples were prepared at a concentration of 100 µg/mL and transferred into capillary tubes (ø 300 µm, 10 mm) to avoid significant thermal convection of solvent during excitation. Each sample was then imaged in real time upon 808 nm laser irradiation of the laser beam focused in a ~15 × 50 µm waist inside the tube. Saved sequences of thermal images were processed by a FLIR camera software to plot the change in maximum temperature of the sample over time.

## 3. Results and Discussion

### 3.1. Physical Characterization

**Figure 2:**
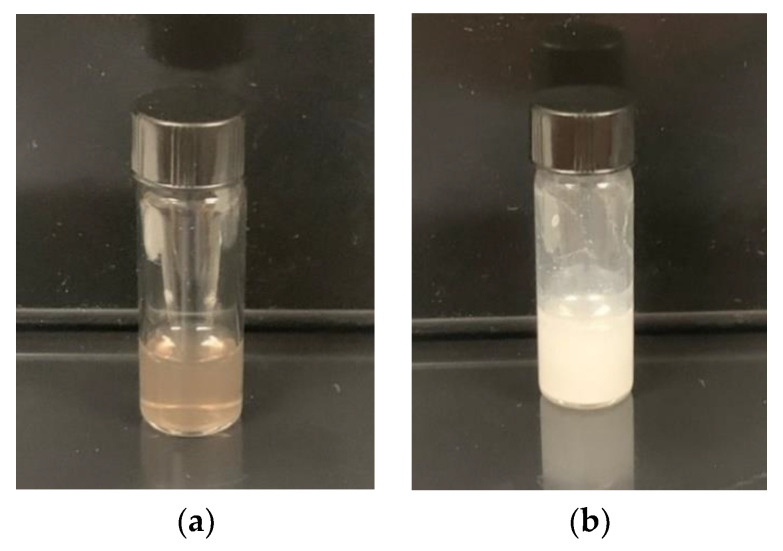
Colloidal solution of (**a**) Bi nanoparticles (NPs) produced from fs LAA and (**b**) Bi nanosheets after fs LAW.

**Figure 3:**
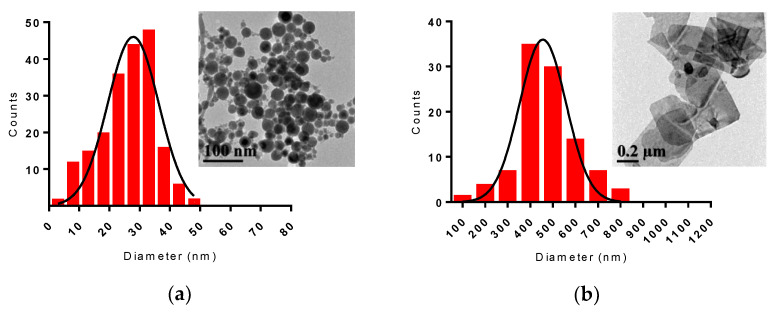
Size distributions and transmission electron microscopy (TEM) images of (**a**) Bi NPs produced from fs LAA and (**b**) Bi nanosheets after fs LAW.

Laser ablation of the Bi target in technical acetone (LAA) and deionized water (LAW) resulted initially in dark-brown colloidal solutions (Figure S1). During the first 5–10 min of the ablation process, there was no visible difference in color of solutions being prepared in any solvents; however, by the end of an ablation experiment (15 min), water-based colloidal solution became substantially turbid, while those prepared in acetone remained clear. NPs prepared by LAW further changed their color to milky-white after approximately 100 min. The final appearances of the colloids synthesized are shown in Figure 2. Transmission electron microscopy (TEM) images taken several days after the preparation revealed significant differences in sizes and morphologies of Bi-based nanomaterials synthesized by LAA and LAW (Figure 3). Bi fs LAA results in spherical NPs with sizes from 5 to 50 nm with an average diameter of 28 ± 4 nm, while large flake-like nanosheets ranging from 185 to 780 nm with an average width of 455 ± 50 nm and a thickness of 10–20 nm are obtained after Bi fs LAW.

**Figure 4:**
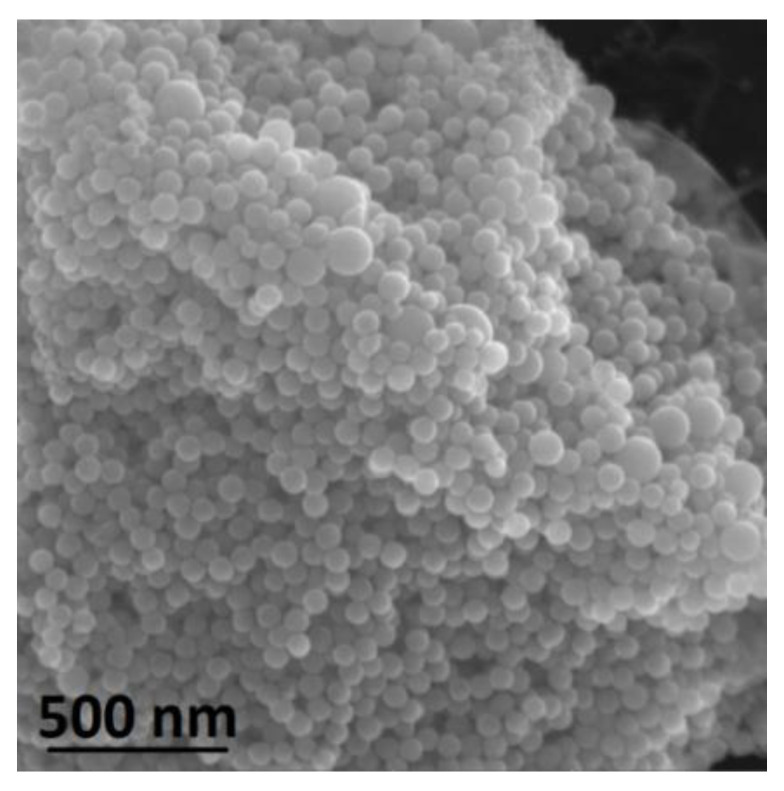
SEM image of Bi NPs produced from fs LAW and re-dispersed in acetone immediately after the synthesis.

We investigated possible reasons for such profound differences in morphology of the nanomaterials prepared by fs laser ablation in different solvents. Scanning electron microscopy (SEM) images of Bi LAW, performed immediately after the synthesis and several days after, demonstrated that the synthesized nanosheets seemed to grow over time and develop a more crystalline shape (Figure S2). No spherical NPs were observed in the SEM images of the Bi sample after LAW; however, relatively long sample preparation time for electron imaging could be a reason for the absence of observed spherical NPs in the sample. To exclude this possibility, we quickly changed the solvent after Bi LAW from deionized water to acetone by centrifugation (1 min, 10,000 RCF). In this case, we observed spherical NPs with a narrow size distribution and a mean diameter of 50 nm (Figure 4). We noticed that the presence of water played a decisive role in the final morphology of synthesized nanomaterials; even the addition of 10 µL of deionized water to 1 mL of sample Bi NPs prepared by LAA resulted in a change morphology from spherical NPs to flake-like nanosheets.

Good colloidal stability of laser-synthesized nanomaterials is dictated by the electrical charging of the material during the ablation process and the related electrostatic stabilization. According to our ζ—potential measurements, the surface potential of Bi NPs prepared by fs LAA was −20 meV, which coincides with the stability threshold for colloidal solutions and is consistent with the observed stability of our sample over time. Not surprisingly, the nanosheets produced as result of Bi fs LAW precipitated out over time; despite their ζ—potential of −40 meV, their large size results in poor colloidal stability [28]. 

**Figure 5:**
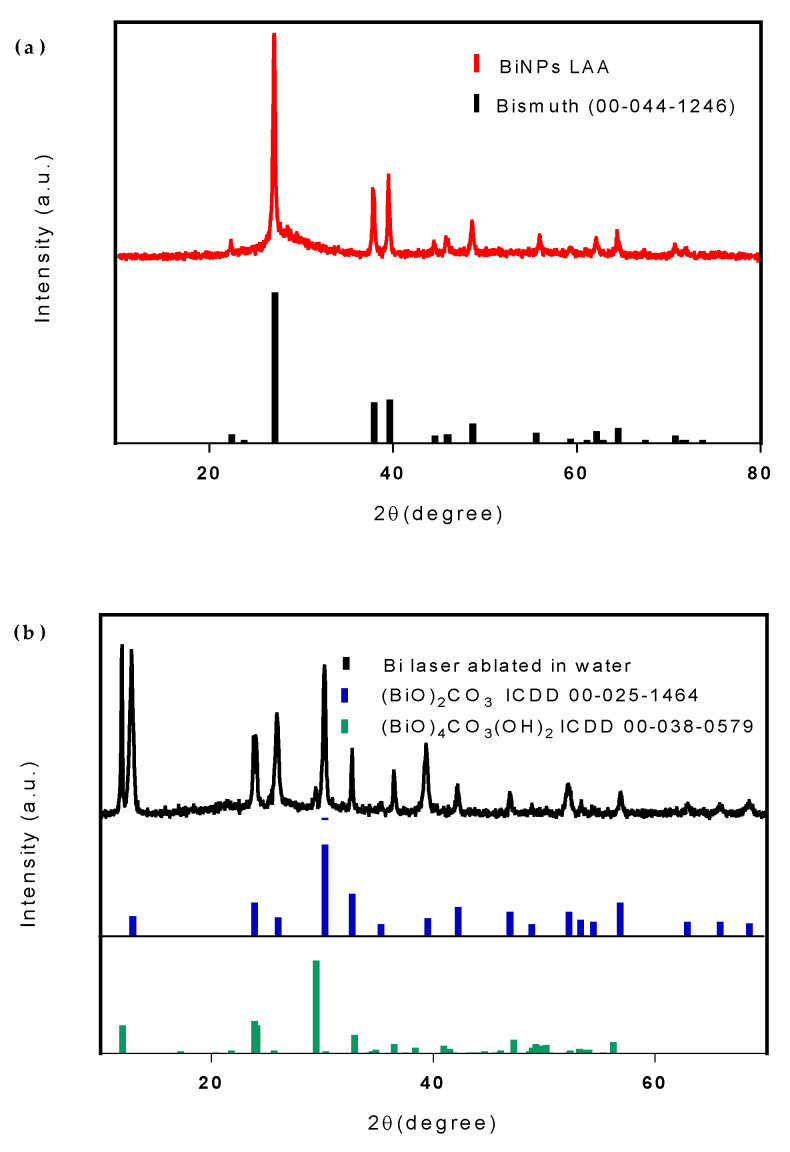
X-ray diffraction (XRD) patterns of (**a**) Bi NPs produced from fs LAA and (**b**) Bi nanosheets after fs LAW.

The X-ray diffraction (XRD) pattern of the Bi nanoparticles generated by fs LAA can be seen in Figure 5a. All the peaks can be indexed with the reference data for Bi metal (ICDD, No. 00-44-1246) with lattice parameters, a = 4.547 Å and c =11.862 Å; no impurity phase is present, indicating LAA generates pure bismuth metal nanoparticles. The XRD pattern of the nanosheets generated by Bi fs LAW can be seen in Figure 5b. Most of the diffraction lines are indexed with the reference data for (BiO)_2_CO_3_ (ICDD No. 00-025-1464). Some peaks characteristic of (BiO)_4_CO_3_(OH)_2_ (ICDD 00-038-0579) were also observed at 11.99, 29.45, 36.50, and 50.19°. This suggests that Bi LAW results in nanosheets containing a combination of both (BiO)_2_CO_3_ and (BiO)_4_CO_3_(OH)2 [29].

**Figure 6:**
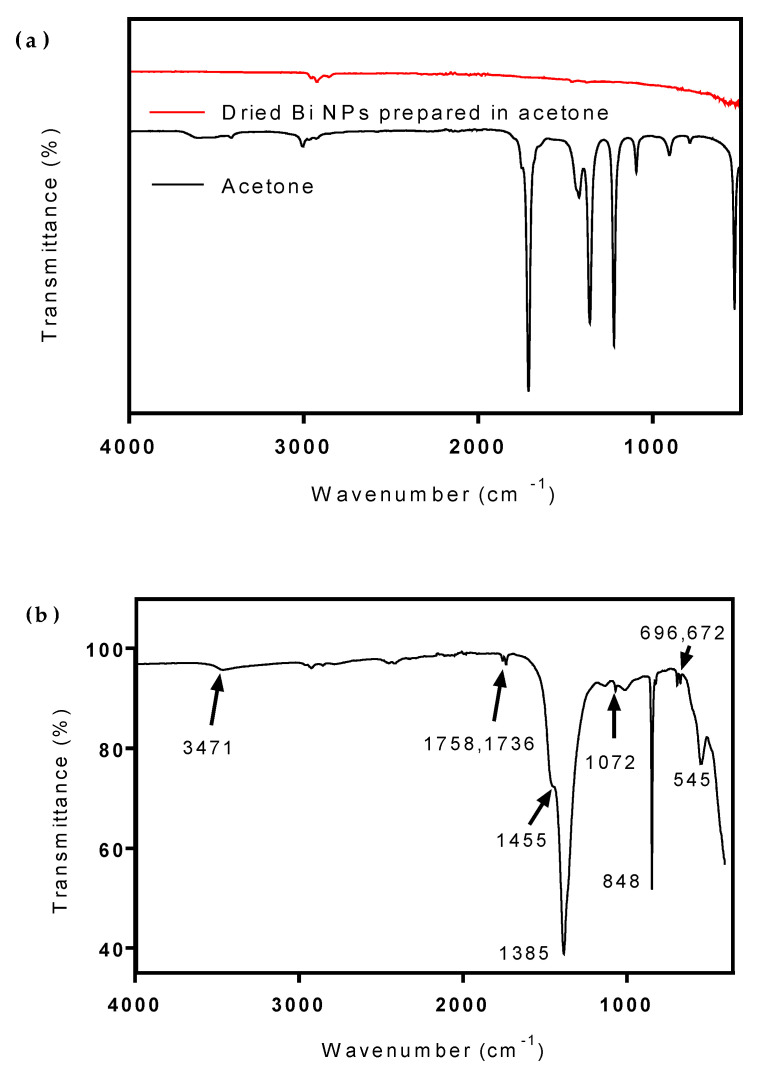
Fourier-transform infrared (FTIR) spectra of (**a**) Bi NPs produced from fs LAA and (**b**) Bi nanosheets after fs LAW.

The FTIR spectrum of Bi fs LAA can be seen in Figure 6a. No characteristic peaks from the Bi NPS are detected, most likely due to their infrared inactivity. The FTIR spectrum of Bi fs LAW can be seen in Figure 6b. The CO_3_^2−^ ion, with a point group symmetry of *D*_3h_, has four internal vibrations, which are all observed in this spectrum. The symmetric stretching mode (*ν*_1_) is attributed to the medium weak absorption band observed at 1072 cm^−1^. The strong absorption band at 1385 cm^−1^ and the shoulder seen at 1458 cm^−1^ can be assigned to the anti-symmetric stretching mode (*ν*_3_) of the CO_3_^2−^ group. The band observed at 848 cm^−1^ is attributed to the out-of-plane bending mode (*ν*_2_), while the bands at 696 and 672 cm^−1^ can be attributed to the in-plane deformation mode (*ν*_4_) of the coordinated CO_3_^2−^ group [30]. The strong absorption band that appears at 545 cm^−1^ is representative of the stretching modes of the Bi–O bonds in (BiO)_2_CO_3_. This supports the hypothesis that Bi LAW produces (BiO)_2_CO_3_ nanosheets. The weakly observed O–H stretch at 3475 cm^−1^ also suggests the possible presence of (BiO)_4_CO_3_(OH)_2_ or uncoordinated water left over after drying [31,32]

**Figure 7:**
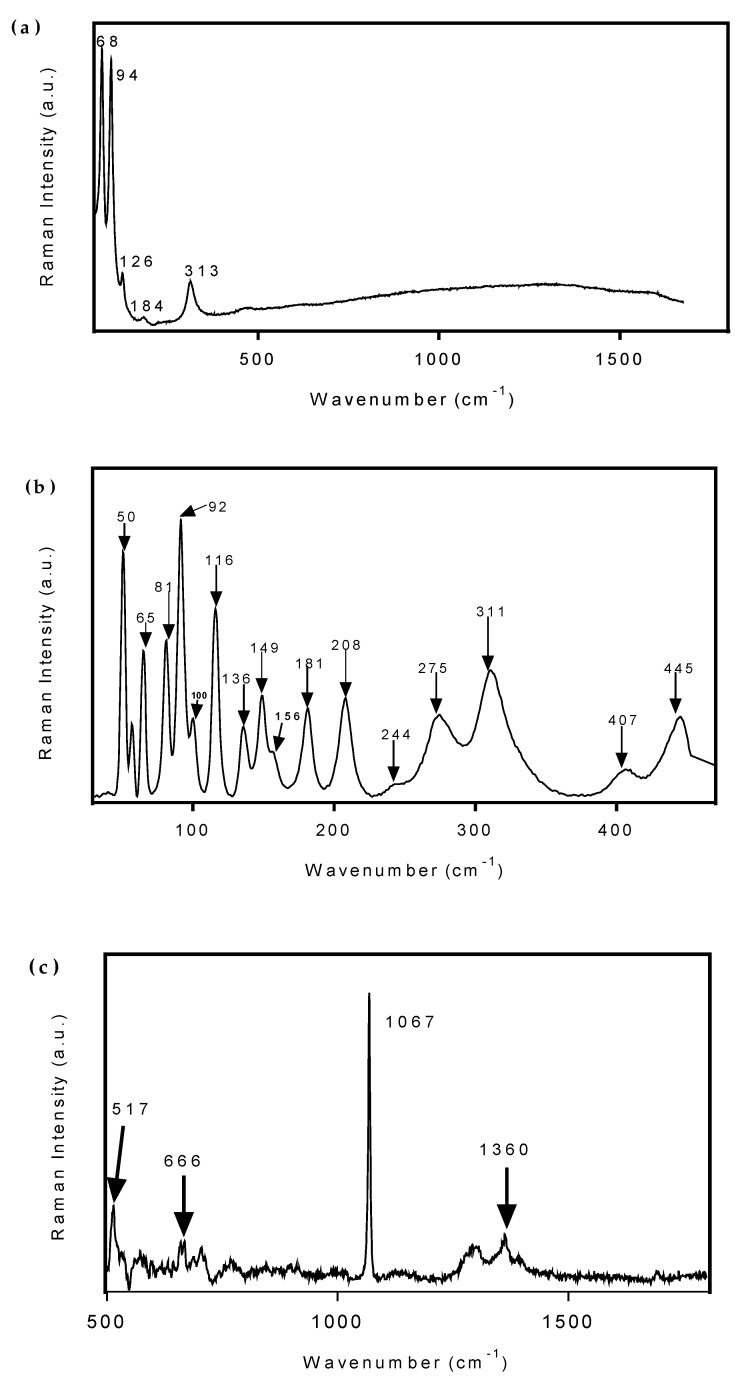
Raman spectra of (**a**) Bi NPs produced from fs LAA and (**b**,**c**) Bi nanosheets after fs LAW.

The Raman spectrum of Bi fs LAA can be seen in Figure 7a. The Raman bands located at 68 and 94 cm^−1^ can be assigned to first order E_g_ and A_1g_ phonon modes, respectively, while a weak second order band is visible at 184 cm^−1^. The bands at 126 and 313 cm^−1^ correspond to unique Bi–O stretches, which are attributed to the β-phase Bi_2_O_3_ [33]. Bi_2_O_3_ impurities were observed in the Raman spectrum but not in the FTIR spectrum or the XRD pattern. This is consistent with laser irradiation-induced oxidation of the bismuth surface, as previously demonstrated by Lewis et al. They demonstrated that for sufficiently large power densities an oxidation reaction occurs, followed by a rearrangement into β-Bi_2_O_3_ [34]. The Raman spectra of Bi fs LAW can be seen in Figure 7b,c. The Raman bands below 600 cm^−1^ are consistent with the lattice and Bi–O vibrations previously reported for (BiO)_2_CO_3_ and (BiO)_4_CO_3_(OH)_2_ [35]. The two bands observed in this spectrum, 244 and 275 cm^−1^, are not seen in the Raman spectrum of pure (BiO)_2_CO_3_ and have been previously been assigned to the concerted motions of the coordinated OH groups [35]. The strong band at 1067 cm^−1^ is representative of the *ν*_1_ mode of the coordinated CO_3_^2−^ group, while the bands at 1360 and 666 cm^−1^ are attributed to the *ν*_4_ and *ν*_3_ modes, respectively. The *ν*_2_ mode is not Raman active and therefore not observed [35]. These spectra support the conclusion that Bi fs LAW results in a combination of (BiO)_2_CO_3_ and (BiO)_4_CO_3_(OH)_2_. 

**Figure 8:**
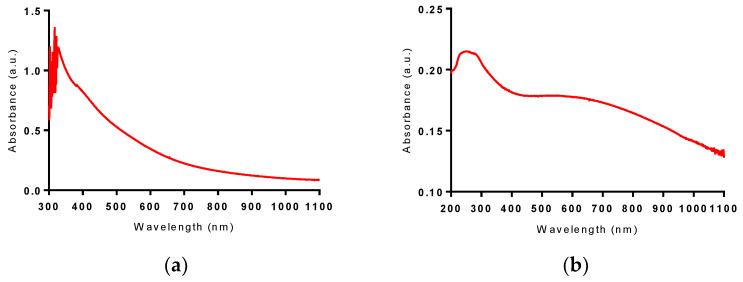
VIS-NIR absorbance spectrum of (**a**) Bi NPs produced from fs LAA and UV-VIS-NIR absorbance spectrum of (**b**) Bi nanosheets after fs LAW.

UV/VIS/NearIinfrared(NIR)spectroscopy was used to characterize the optical absorption properties of the materials. The absorbance spectra of Bi LAA and Bi LAW can be seen in Figure 8; it is featureless and continuous above 400 nm. This spectrum is representative of previously reported pure elemental Bi NPs; however, they typically have an absorbance peak below 300 nm [36,37]. This peak cannot be observed in the Bi LAA sample due to the high absorbance of acetone below 300 nm [27]. The absorbance of Bi LAW lies mainly below 400 nm in the ultraviolet region, with a maximum absorbance between 230 and 280 nm; this is consistent with that seen for (BiO)_2_CO_3_ [38,39].

**Figure 9:**
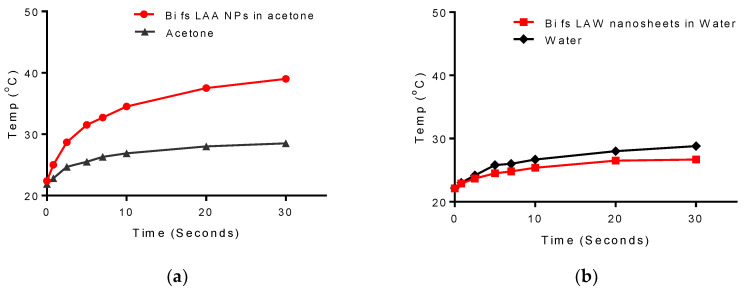
Photothermal heating of (**a**) Bi NPs produced from fs LAA and (**b**) Bi nanosheets after fs LAW, using an 800-nm excitation source at 1-watt power.

To determine the potential of the synthesized Bi NPs and nanosheets to be used for localized heating under NIR (808 nm) excitation, we studied the dynamics of thermal heating for both nanomaterials. The results of this study (Figure 9) show that elemental Bi NPs, but not Bi nanosheets, demonstrated a significant increase in temperature after 30 s exposure to the NIR excitation. The initial rise in temperature of the elemental Bi NPs upon exposure is approximately 2 °C/ second for the first 5 s reaching a temperature of 31.5 °C. This rate levels off to approximately 0.4 °C/second over the remaining 25 s, leading to a temperature maximum of 39 °C, for an overall increase of 16.5 °C. The Bi nanosheets only demonstrate a 4.5 °C increase in temperature over the same 30 s exposure period. This result shows the relative merit of elemental Bi NPs for near IR photothermal therapy and as a contrast agent for near IR photoacoustic imaging. 

### 3.2. Stability Studies

**Figure 10:**
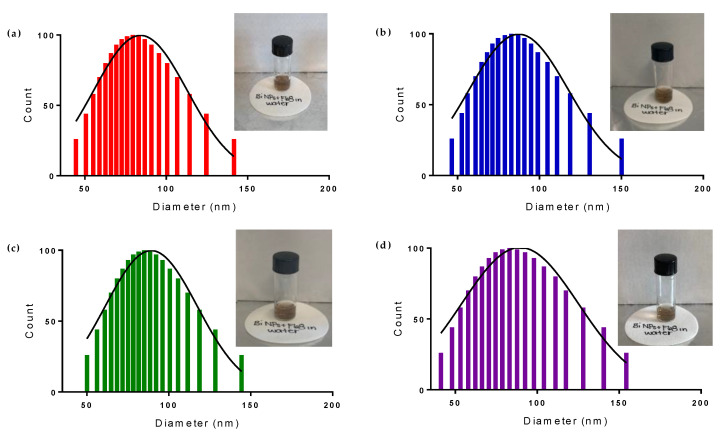
Colloidal solutions and size distributions, determined by Dynamic Light Scattering (DLS), of pluronic^®^ F68 coated Bi NPs (**a**) immediately after, (**b**) 5 days after, (**c**) 10 days after, and (**d**) 14 days after transfer to water.

To create a stable dispersion of Bi NPs in water, Pluronic^®^ F68 was used to coat the surface and prevent the conversion of the NPs from pure Bismuth metal to oxide or carbonate compounds. The stability of the Bi NPs in water was evaluated by observing changes in color, hydrodynamic diameter, and Raman spectra of coated elemental Bi NP water dispersion over 14 days. Figure 10 demonstrates the color stability of the Bi NPs dispersion over time. The uncoated Bi NP dispersion in water changes from brown to white after 1 day (Figure S3), while the coated Bi NP dispersion maintains its brown color for more than 14 days. Additionally, aside from a slight increase in the hydrodynamic diameter due to the presence of the polymer surface coating, the coated Bi NPs maintain their size for more than 14 days, while a dramatic increase in the hydrodynamic diameter, from ~50 to ~400 nm, is observed in the uncoated Bi NPs in water after just 1 day (Figure 10 and Figure S3). This suggests that the coated Bi NPs are not converting to oxide or carbonate compounds, as is occurring with the uncoated Bi NPs.

**Figure 11:**
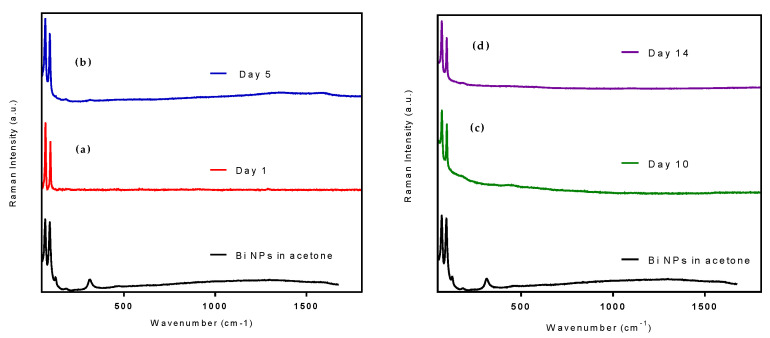
Raman spectra of Pluronic ^®^ F68 coated Bi NPs (**a**) immediately after, (**b**) 5 days after, (**c**) 10 days after, and (**d**) 14 days after transfer to water, compared with as-synthesized Bi NPs in acetone.

To confirm that the retention of color and size are indicative of the stability of the coated Bi NPs in water, we simultaneously evaluated the chemical composition of the NPs using Raman spectroscopy (Figure 11). The strong Raman bands located at 68 and 94 cm^−1^ in all four samples confirm the retention of pure Bi metal NPs after transfer to water using Pluronic^®^ F68. The absence of the strong band at 1067 cm^−1^, representative of the *ν*_1_ mode of CO_3_^2−^, confirms that the sample of coated Bi NPs in water contains no CO_3_^2^. Additionally, the absence of the bands at 126 and 313 cm^−1^_,_ which correspond to the unique Bi–O stretches of β-phase Bi_2_O_3_, confirms that we have successfully prepared stable elemental Bi NPs in water using this coating method. This is also supported by the XRD pattern of the coated NPs (Figure S4), which can be indexed with the reference data for Bi metal (ICDD, No. 00-44-1246) without the presence of any impurity phase. This reinforces the conclusion that the F68 coated Bi NPs are not undergoing the conversion to (BiO)_2_CO_3_ when transferred to water. 

**Figure 12:**
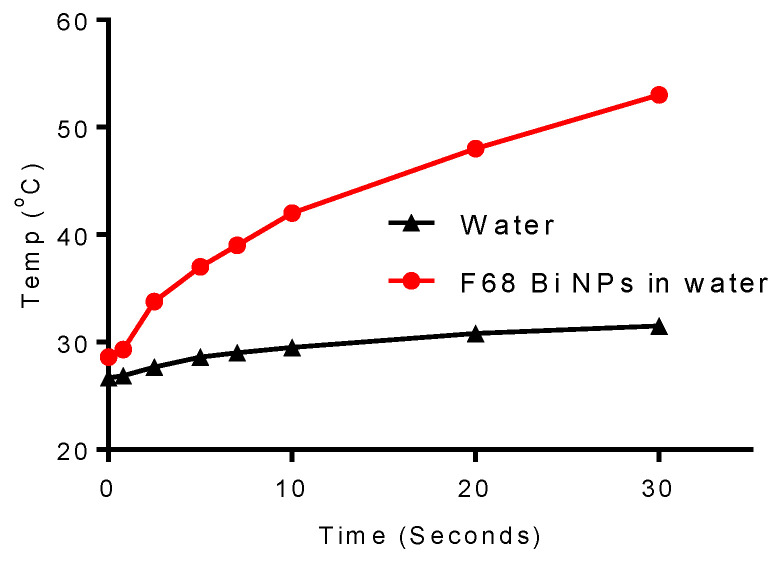
Photothermal heating of Pluronic^®^ F68 coated Bi NPs, 10 days after transfer to water, using an 800-nm excitation source at 1-watt power.

Lastly, we wanted to evaluate the dynamics of thermal heating for the Pluronic ^®^ F68 coated Bi NPs in water to confirm that this transfer does not reduce their localized heating potential. The results of this study (Figure 12) show that the coated Bi NPs demonstrated a significant increase in temperature, greater than that of the NPS in acetone, after 30 s exposure to the NIR excitation. Upon exposure, the initial rise in temperature of the coated Bi NPs in water is approximately 2 °C/s for the first 5 s reaching a temperature of 37 °C. This increase rate slows down to 1.0 °C/s over the next 5 s, and levels off at 0.5 °C/second for the remaining 20 s to finally yield a temperature maximum of 53 °C, for an overall increase of 24.4 °C. This suggests that the coated NPs transferred to water will be effective for use in photothermal therapy in vivo as well as for photoacoustic imaging. 

## 4. Conclusions

We elaborated methods of femtosecond laser ablation from a Bi target in liquid ambient to fabricate Bi-based nanomaterials for biomedical applications. We showed that Bi-based NPs prepared in deionized water rapidly convert into 400–500 nm flake-like nanosheets composed of bismuth subcarbonates, while the NPs prepared in acetone present stable solutions of crystalline elemental Bi NPs having the mean size of 20–40 nm and a low size-dispersion. We also showed that Bi NPs prepared in acetone can be transferred to water via coating with Pluronic^®^ F68. After such a transfer, aqueous NPs solutions demonstrated a high stability (stability characteristics were recorded for at least two weeks) and are very suitable for use in biological systems. It was also found that the NPs could exhibit remarkable absorption in the infrared range, making them a promising nanoplatform for photothermal therapy. Based on obtained physico-chemical characteristics and exceptional purity, laser-synthesized NPs promise a major advancement of current methods for photo/radiotherapies.

## Supplementary Materials

The following are available online at https://www.mdpi.com/2079-4991/10/8/1463/s1, Figure S1. Colloidal solutions of Bi-based nanostructures immediately after (a) fs LAW and (b) fs LAA, Figure S2. SEM images of Bi nanosheets prepared by LAW (a) immediately after synthesis and (b) several days after, Figure S3. Colloidal solutions and size distributions, determined by DLS, of uncoated Bi NPs (a) immediately after and (b) 1 day after transfer to water, Figure S4. X-Ray Diffraction (XRD) patterns of Pluronic® F68 coated Bi NPs 10 days after transfer to water.
