# Supplementary material for: Laser-Ablative Synthesis of Stable Aqueous Solutions of Elemental Bismuth Nanoparticles for Multimodal Theranostic Applications
Source: Nanomaterials (Basel). 2020 Jul 26;10(8):1463. doi: 10.3390/nano10081463 (PMC7466601; doi:10.3390/nano10081463)
Supplement: Supplementary file 1 [file nanomaterials-10-01463-s001.pdf]

Laser-Ablative Synthesis of Stable Aqueous Solutions of Elemental Bismuth Nanoparticles for Multimodal Theranostic Applications

Julia C. Bulmahn [1], Gleb Tikhonowski [2], Anton A. Popov [2], Andrey Kuzmin [1], Sergey M. Klimentov [2], Andrei V. Kabashin [2,3,*] and Paras N. Prasad [1,2,*]

[1] Department of Chemistry and Institute for Lasers, Photonics, and Biophotonics, University at Buffalo, The State University of New York, Buffalo, NY 14260, USA; juliabul@buffalo.edu (J.C.B.); ankuzmin@buffalo.edu (A.K.)

[2] Bionanophotonic Lab., Institute of Engineering Physics for Biomedicine (PhysBio), National Nuclear Research University MEPHI, Moscow 115409, Russia; gtikhonowski@gmail.com (G.T.); AAPopov@mephi.ru (A.A.P.); kliment-61@mail.ru (S.M.K.)

[3] LP3, Aix Marseille University, CNRS, 13288 Marseille, France; kabashin@lp3.univ-mrs.fr

* Correspondence: pnprasad@buffalo.edu (P.N.P.); kabashin@lp3.univ-mrs.fr (A.V.K.)

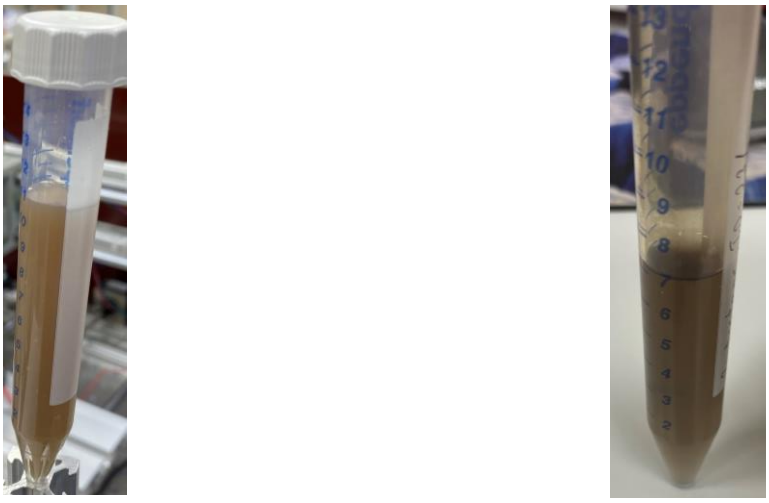

(a) (b)

Figure S1. Colloidal solutions of Bi-based nanostructures immediately after (**a**) fs LAW and (**b**) fs LAA.

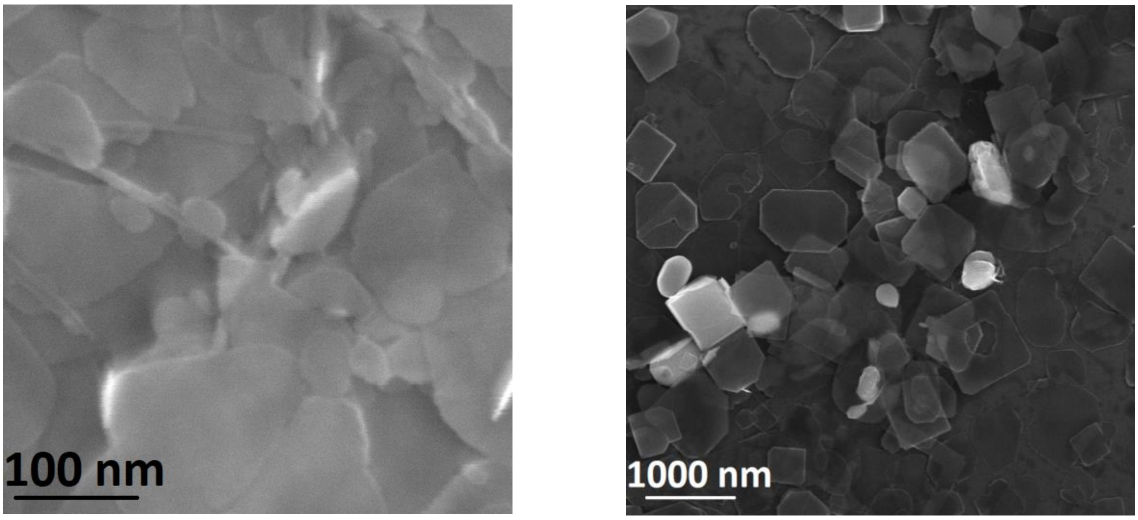

(a) (b)

Figure S2. SEM images of Bi nanosheets prepared by LAW (**a**) immediately after synthesis and (**b**) several days after.

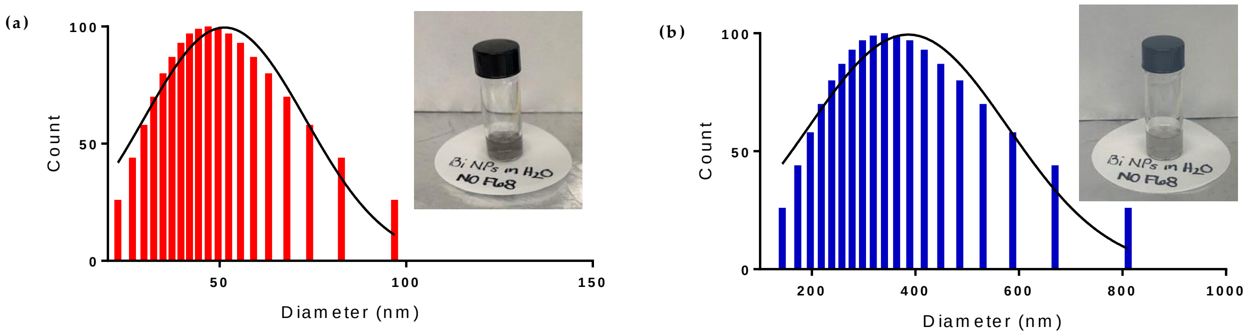

Figure S3. Colloidal solutions and size distributions, determined by DLS, of uncoated Bi NPs (**a**) immediately after and (**b**) 1 day after transfer to water.

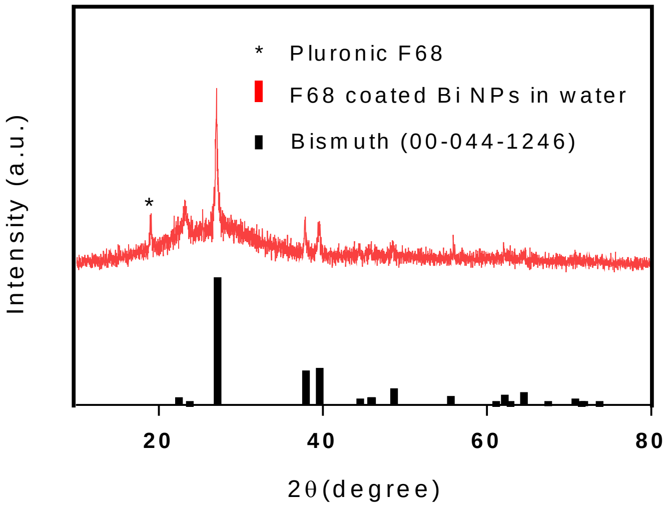

Figure S4. X-Ray Diffraction (XRD) patterns of Pluronic® F68 coated Bi NPs 10 days after transfer to water.
